# Comparative effectiveness of mesenchymal stem cell versus bone-marrow mononuclear cell transplantation in heart failure: a meta-analysis of randomized controlled trials

**DOI:** 10.1186/s13287-024-03829-7

**Published:** 2024-07-06

**Authors:** Alireza Hosseinpour, Jahangir Kamalpour, Niloofar Dehdari Ebrahimi, Seyed Alireza Mirhosseini, Alireza Sadeghi, Shahin Kavousi, Armin Attar

**Affiliations:** 1 Department of Cardiovascular Medicine, Shiraz University of Medical Sciences, Shiraz, Iran; 2 School of Medicine, Shiraz University of Medical Sciences, Shiraz, Iran; 3 Transplant Research Center, Shiraz University of Medical Sciences, Shiraz, Iran; 4 MD-MPH Department, School of Medicine, Shiraz University of Medical Sciences, Shiraz, Iran

**Keywords:** Chronic heart failure, Stem cell therapy, Mesenchymal stem cells, Bone-marrow mononuclear cells

## Abstract

**Background:**

There is no clear evidence on the comparative effectiveness of bone-marrow mononuclear cell (BMMNC) vs. mesenchymal stromal cell (MSC) stem cell therapy in patients with chronic heart failure (HF).

**Methods:**

Using a systematic approach, eligible randomized controlled trials (RCTs) of stem cell therapy (BMMNCs or MSCs) in patients with HF were retrieved to perform a meta-analysis on clinical outcomes (major adverse cardiovascular events (MACE), hospitalization for HF, and mortality) and echocardiographic indices (including left ventricular ejection fraction (LVEF)) were performed using the random-effects model. A risk ratio (RR) or mean difference (MD) with corresponding 95% confidence interval (CI) were pooled based on the type of the outcome and subgroup analysis was performed to evaluate the potential differences between the types of cells.

**Results:**

The analysis included a total of 36 RCTs (1549 HF patients receiving stem cells and 1252 patients in the control group). Transplantation of both types of cells in patients with HF resulted in a significant improvement in LVEF (BMMNCs: MD (95% CI) = 3.05 (1.11; 4.99) and MSCs: MD (95% CI) = 2.82 (1.19; 4.45), between-subgroup *p* = 0.86). Stem cell therapy did not lead to a significant change in the risk of MACE (MD (95% CI) = 0.83 (0.67; 1.06), BMMNCs: RR (95% CI) = 0.59 (0.31; 1.13) and MSCs: RR (95% CI) = 0.91 (0.70; 1.19), between-subgroup *p* = 0.12). There was a marginally decreased risk of all-cause death (MD (95% CI) = 0.82 (0.68; 0.99)) and rehospitalization (MD (95% CI) = 0.77 (0.61; 0.98)) with no difference among the cell types (*p* > 0.05).

**Conclusion:**

Both types of stem cells are effective in improving LVEF in patients with heart failure without any noticeable difference between the cells. Transplantation of the stem cells could not decrease the risk of major adverse cardiovascular events compared with controls. Future trials should primarily focus on the impact of stem cell transplantation on clinical outcomes of HF patients to verify or refute the findings of this study.

**Supplementary Information:**

The online version contains supplementary material available at 10.1186/s13287-024-03829-7.

## Background

Heart failure represents a significant global health burden, affecting a substantial proportion of the worldwide population [1]. Although significant progress has been made in the development of pharmacological interventions [2] and implantable cardiac devices [3, 4], the overall clinical outcomes for patients diagnosed with heart failure remain suboptimal, underscoring the need to investigate innovative therapeutic modalities. Stem cell-based therapies are novel approaches with the potential to improve the morbidity and mortality associated with heart failure [5]. It is believed that stem cell transplantation can result in higher regional blood flow, angiogenesis, and improved cardiac function, which is mediated by increased paracrine signaling pathways obtained from higher expression of interleukin-1β (IL-1β), tissue necrosis factor-α (TNF-α), and vascular endothelial growth factor (VEGF) [6–8]. Many types of stem cells have been frequently used in the past few years to improve the clinical outcomes of patients with heart failure (HF). Mesenchymal stromal cells (MSCs) [9] and bone marrow-derived mononuclear cells (BMMNCs) [10] transplantation have both emerged as possibly important therapies for HF patients due to their potential for cardiac repair. MSCs, multipotent stromal cells that can differentiate into a variety of cell types, including cardiomyocytes, have shown potential in preclinical and clinical studies [11]. Using the current criteria for isolation, MSCs produce heterogenous, non-clonal cultures, comprising stromal cells with varying multipotential capabilities, along with committed progenitors and differentiated cells. These cultures demonstrate a wide range of differentiation potentials resulting in a diverse array of cell types within the cultures [12, 13]. On the other hand, BMMNCs, a heterogeneous population of cells, including hematopoietic stem cells and endothelial progenitor cells, have also demonstrated beneficial effects on cardiac function and structure [14, 15]. Although a previous meta-analysis has compared BMMNCs with MSCs in patients with acute myocardial infarction, a clear comparison between the two types of therapies in chronic heart failure has not been established. This meta-analysis was conducted to determine and compare the cardiovascular outcomes and echocardiographic indices of MSCs and BMMNCs therapies in heart failure.

## Methods

This systematic review was reported based on the Preferred Reporting Items for Systematic Reviews and Meta-Analyses (PRISMA) guidelines [16]. Prospective protocol registration was done with the registration ID of CRD42024504239.

### Search sources and strategies

An extensive search of the literature was carried out in three different online databases including PubMed, Scopus, and Embase Library to find the eligible studies published from database inception up until February 20th, 2024. No time frame or restriction was placed on the search results. The search strategy of our study included the relevant keywords mentioned in Table S1.

### Study selection and risk of *bias*

First, the duplicate records were removed and they were subsequently imported into the Rayyan web-based tool for managing systematic reviews [17]. Two reviewers (ND and SM) autonomously assessed the records based on their titles and abstracts. Subsequently, full texts were obtained for each study to undergo screening based on the eligibility criteria. Discrepancies were addressed through discussion with a third author (AH) until consensus was reached. Eligible studies were required to meet the following criteria: (a) randomized controlled trials (RCTs), (b) patients with chronic heart failure or cardiomyopathy (ischemic or non-ischemic), (c) administration of BMMNCs or MSCs in at least one trial arm, (d) presence of one or more control arms who were treated with standard therapy with or without placebo injection, and (e) reporting clinical outcomes or echocardiographic indices. Studies with potential overlapping population were identified and the study with the larger sample size was included.

For the present study, the primary outcome of interest was major adverse cardiovascular events (MACE), all-cause mortality, and hospitalization for heart failure at the longest available follow-up. The secondary outcomes were echocardiographic indices (left ventricular ejection fraction (LVEF), left ventricular end-diastolic volume (LVEDV), and left ventricular end-systolic volume (LVESV)), 6-min walk test (6MWT), and B-type natriuretic peptide (BNP).

The reviewers (ND, SM) extracted data into a predefined form within Microsoft Excel Spreadsheet Software. A third author (AH) cross-checked the data accuracy and resolved any extraction discrepancies through discussion. For each study, the following data were extracted: (a) study characteristics (first author, publication year, trial name, and country), (b) subject characteristics (sample size, type of heart failure, and baseline demographics of treatment and control group), (c) intervention specifics (dosage and type of stem cells administered), (d) clinical outcomes (MACE, all-cause mortality, and rehospitalization), (e) echocardiographic indices (baseline, final measurements, changes from baseline during follow-up period) including LVEF, LVEDV, and LVESV, and (f) the baseline and follow-up values of 6MWT and BNP. The clinical outcomes and echocardiographic indices were extracted at the longest available follow-up.

The quality assessment of the eligible studies was conducted using the Cochrane Collaboration's tool for evaluating bias in randomized trials [18]. The included RCTs each underwent quality assessment and were assigned to categories of high, low, or some concerns risk of bias within various domains. A total risk of bias was then assigned to each of the studies based on the risk of bias within each domains. Risk of Bias plots were generated using “Risk-of-bias visualization (robvis)” R package [19].

### Data synthesis and ICEMAN tool assessment

The analyses performed for the present study was undertaken in R software version 4.3.2 with “meta” and “metaphor” packages being used. For binary outcomes, the number of events and total sample size were used to perform the analysis and generate a risk ratio (RR) and its corresponding 95% confidence interval (CI) with Mantel–Haenszel method being used. For continuous outcomes, based on the pre-post intervention data, a mean difference (MD) and standard error of the MD were calculated and inserted for the analysis with inverse variance method. We used both BNP and N-terminal proBNP (NT-proBNP) levels and since there is difference in measurement scales between the two parameters, we used standardized mean difference (SMD) to pool the results. For both types of outcomes, the random effects model was used. The outcomes were analyzed for both an overall effect and subgroup difference. The studies were stratified based on the type of the stem cell used (BMMNC vs. MSC) to test for any potential difference between the subgroups in all the analyses. A subgroup analysis was also conducted to investigate the difference between the routes of injection. For better evaluation of potential differences between the two types of stem cells, we used Instrument to assess the Credibility of Effect Modification Analyses (ICEMAN) in meta-analysis of randomized controlled trials. It is consisted of a total of eight questions to assess the credibility of the results and for each outcome, a total rating is given based on the answers to each questions [20]. The *p*-values were reported for the overall effect and subgroup differences. The statistical significance was met if the pooled estimate did not cross the null zone.

## Results

### Characteristics of the included studies

A total of 20 randomized controlled trials involving BMMNCs (622 participants receiving stem cell and 495 controls) [14, 21–39] and 17 trials [24, 40–55] with MSCs (927 receiving stem cells and 757 controls) were considered eligible for meta-analysis. The search of Scopus, PubMed, and Embase library yielded a total of 7316 citations. After adjusting for duplicates, 5393 records remained. Upon abstract review and screening, it was determined that 5147 of these studies did not fulfill the established criteria and were consequently excluded. The full-text of the remaining 246 citations underwent a detailed examination. It was determined that 211 studies did not meet the inclusion criteria as described and were subsequently discarded.

**Fig. 1:**
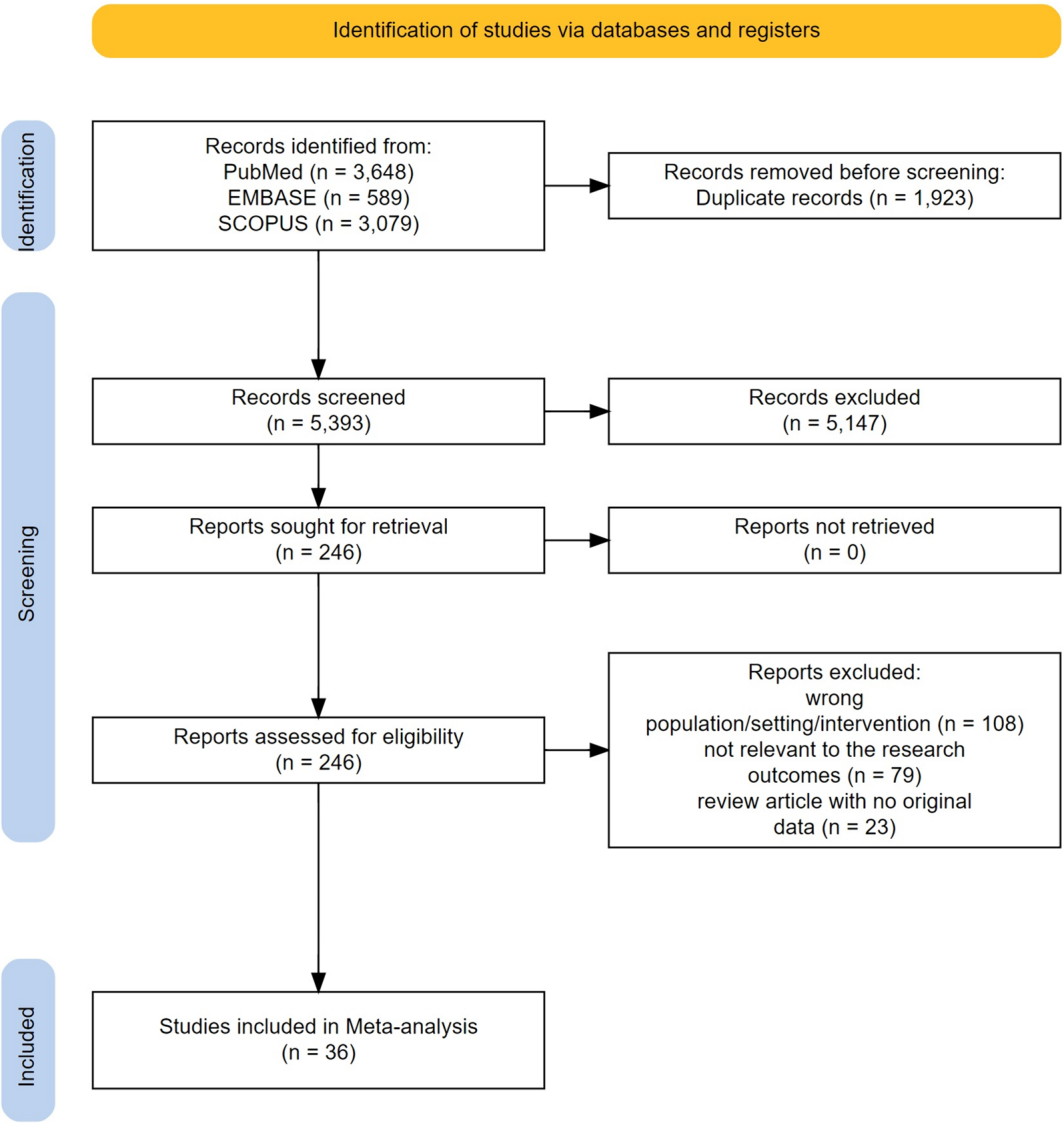
PRISMA flowchart demonstrating the search and screening process

The final selection for the systematic review and meta-analysis comprised 36 studies (one study including data for both MSCs and BMMNCs [24]), all of which were randomized controlled trials (Fig. 1). In the included MSCs studies, the origin of the cells was diverse: Seven studies [24, 41–43, 45, 46, 53] utilized autologous bone marrow, while four studies [44, 47, 48, 54] employed allogenic bone marrow. MSCs derived from adipose tissue were used in three studies [49–51], and three studies used allogenic MSCs from umbilical cord [40, 52, 55]. The sample size ranged from 16 to 537 patients with HF. The lowest and highest LVEF among groups were 16.2 ± 6.0 and 54.0 ± 8.0, respectively. Table 1 provides an overview of the data pertaining to each individual study. This includes information such as the countries where the studies were conducted, the sample sizes used in each study, and the mean age range of the participants. Additionally, it also details the type of cells used in each study, among other relevant factors.

**Table 1:** General characteristics of the included trials

Study	Country	Type of cell	Sample size		Mean age			% of Male		Baseline LVEF		Route of delivery
			Inv	Ctrl	Inv	Ctrl		Inv	Ctrl	Inv	Ctrl	
Assmus [14]	Germany	BMMNCs	62	39	61.0 ± 12.2	61.5 ± 9.9		82	85	34.4 ± 10.1	31.1 ± 12.2	Intracoronary
Choudhury [21]	United Kingdom	BMMNCs	30	30	63.7 ± 9.5	61.6 ± 10.8		97	97	30.2 ± 8.9	30.3 ± 7.7	Intracoronary or Intramyocardial
Duan [22]	USA	BMMNCs	24	18	57.9 ± 8.5	56.6 ± 9.1		96	94	ND	ND	During CABG
Hamshere [23]	United Kingdom	BMMNCs	15	15	57.7 ± 12.3	54.9 ± 10.9		67	60	32.9 ± 16.5	41.7 ± 15.3	Intracoronary
Heldman [24]	USA	BMMNCs	19	10	61.1 ± 8.4	61.3 ± 9.0		90	100	35.9 ± 8.2	36.2 ± 7.4	Transendocardial
Hu [25]	USA	BMMNCs	31	29	56.6 ± 9.7	58.3 ± 8.9		93		23.3 ± 6.0	24.9 ± 6.6	During CABG
Lehtinen [26]	Finland	BMMNCs	20	19	69.3 ± 9.9	69.0 ± 11.2		ND	ND	38.5 ± 9.9	37.7 ± 14.8	Intramyocardial
Mann [27]	Netherlands	BMMNCs	19	20	65.0 ± 7.0	65.0 ± 8.0		100	95	32.6 ± 12.3	29.0 ± 9.7	Intramyocardial
Martino [28]	Brazil	BMMNCs	82	78	51.0 ± 11.1	49.6 ± 11.1		73	68	24 ± 10.8	24.3 ± 9.9	Intracoronary
Mozid [29]	United Kingdom	BMMNCs	34	15	66.8 ± 9.8	66.0 ± 9.0		97	93	28.2 ± 7.0	28.0 ± 8.0	Intracoronary and intramyocardial
Pätilä [30]	Finland	BMMNCs	20	19	65.0 ± 12.8	64.0 ± 9.6		95	95	36.9 ± 12.6	36.0 ± 13.1	Intramyocardial
Perin [31]	USA	BMMNCs	20	10	60.5 ± 6.4	56.3 ± 8.6		80	50	37.3 ± 10.6	39.0 ± 9.1	Transendocardial
Perin [32]	USA	BMMNCs	61	31	63.9 ± 10.9	62.3 ± 8.3		87	94	32.4 ± 9.2	30.2 ± 7.8	Transendocardial
Pokushalov [33]	Russia	BMMNCs	55	54	61.0 ± 9.0	2.0 ± 5.0		87	85	27.8 ± 3.4	26.8 ± 3.8	Intramyocardial
Sant'Anna [34]	Brazil	BMMNCs	20	10	48.3 ± 8.7	51.6 ± 7.8		65	50	21.8 ± 41.2	24.8 ± 4.6	Intramyocardial
Santoso [35]	Hong kong and indonesia	BMMNCs	19	9	58.0 ± 9.9	60.0 ± 5.6		95	100	23.6 ± 8.4	26.8 ± 8.8	Intramyocardial
Seth [36]	India	BMMNCs	41	40	45.0 ± 15.0	49.0 ± 9.0		81	88	22.5 ± 8.3	20.8 ± 9.3	Intracoronary
Trifunović [39]	Serbia	BMMNCs	15	15	53.8 ± 10.1	60.0 ± 6.8		93	93	35.3 ± 3.9	36.5 ± 5.3	Intramyocardial during CABG
Wang [37]	China	BMMNCs	17	16	65.6 ± 4.0	65.5 ± 5.6		45	45	34.0 ± 4.5	34.8 ± 2.9	Intramyocardial
Zhao [38]	China	BMMNCs	18	18	60.3 ± 10.4	59.1 ± 15.7		83	83	35.8 ± 7.3	36.7 ± 9.2	Intramyocardial
Bartolucci [40]	Chile	MSCs	15	15	57.3 ± 10.1		57.2 ± 11.6	80	93	33.5 ± 6.1	31.5 ± 4.9	Intravenous
Bartunek [41]	Belgium	MSCs	21	15	55.7 ± 10.4		59.5 ± 8.0	95	92	27.5 ± 4.7	27.8 ± 4.0	Intramyocardial
Bartunek [42]	Belgium	MSCs	120	151	61.6 ± 8.6		62.1 ± 8.7	89	90	27.3 ± 6.8	28.0 ± 6.0	Intramyocardial
Bolli [43]	USA	MSCs	14	17	54.7 ± 12.8		58.2 ± 11.2	43	24	34.5 ± 2.9	33.3 ± 6.2	Transendocardial
Bolli [44]	USA	MSCs	29	32	61.0 ± 11.1		63.1 ± 8.8	93	97	29.3 ± 5.9	29.7 ± 6.2	Transendocardial
Mathiasen [45]	Denmark	MSCs	40	20	66.1 ± 7.7		64.2 ± 10.6	90	70	28.2 ± 9.3	25.1 ± 8.5	Intramyocardial
Mohyeddin [46]	Iran	MSCs	8	8	49.0 ± 9.4		53.3 ± 6.7	88	75	38.8 ± 13.0	41.9 ± 8.4	Intracoronary and Intramyocardial
Heldman [24]	USA	MSCs	19	11	57.1 ± 10.6		60.0 ± 12.0	95	91	35.7 ± 9.0	28.1 ± 9.8	Transendocardial
Perin [48]	USA	MSCs	45	15	62.2 ± 10.3		62.7 ± 11.2	98	73	31.3 ± 8.6	34.6 ± 6.4	Transendocardial
Perin [47]	USA	MSCs	265	272	62.7 ± 10.9		62.6 ± 10.4	78	78	28.6 ± 6.6	28.6 ± 7.0	Transendocardial
Qayyum [49]	Denmark	MSCs	28	13	65.5 ± 9.7		65.3 ± 8.7	88	100	52.0 ± 8.0	54.0 ± 8.0	Intramyocardial
Qayyum [50]	Denmark	MSCs	54	27	67.0 ± 9.0		66.6 ± 8.1	82	89	34.2 ± 7.9	31.4 ± 7.2	Intramyocardial
Qayyum [51]	Denmark	MSCs	90	43	66.4 ± 8.1		64.0 ± 8.8	93	88	31.6 ± 7.2	32 ± 8.9	Intramyocardial
Ulus [52]	Turkey	MSCs	26	16	61.8 ± 10.0		65.30 ± 6.8	100	100	34.8 ± 4.8	36.2 ± 5.6	Intramyocardial
Xiao [53]	China	MSCs	17	20	51.6 ± 12.2		54.4 ± 11.6	71	70	34.1 ± 3.6	33.7 ± 4.0	Intracoronary
Yau [54]	Canada And USA	MSCs	106	53	55.5 ± 12.3		56.9 ± 11.7	89	89	17.3 ± 5.8	16.2 ± 6.0	Intramyocardial
Zhao [55]	China	MSCs	30	29	52.9 ± 16.3		53.2 ± 11.5	80	65	30.0 ± 4.5	28.0 ± 5.1	Intracoronary

BMMNCs: bone-marrow mononuclear cells, MSCs: mesenchymal stem cells, Inv: intervention, Ctrl: control, LVEF: left ventricular ejection fraction, CABG: coronary artery bypass grafting

### Risk of *bias*

**Fig. 2:**
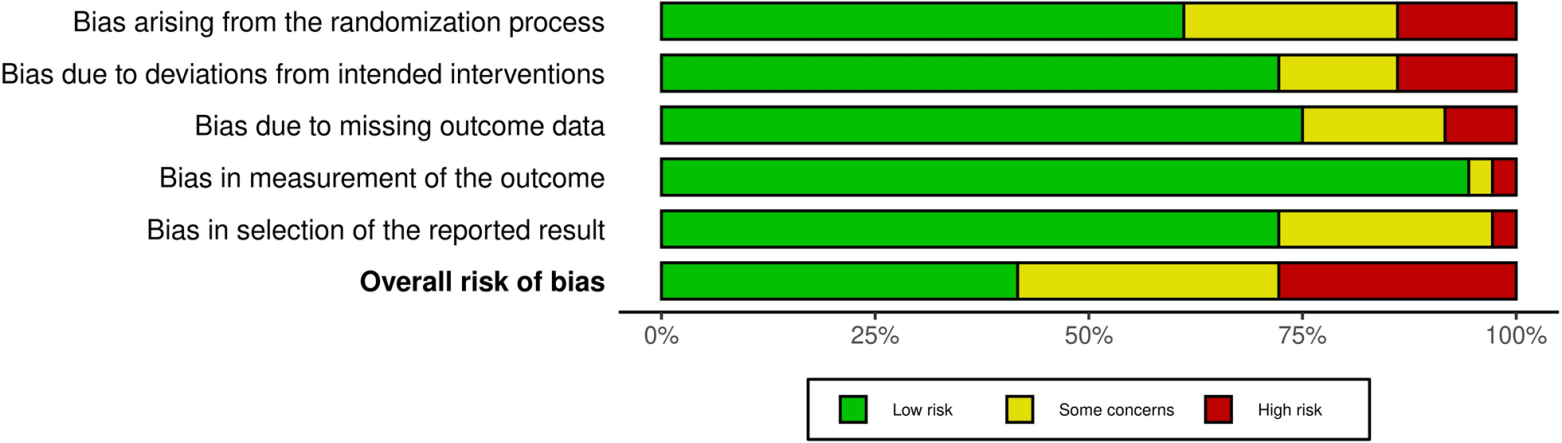
Risk of bias assessment

Utilizing the Cochrane Collaboration’s tool for Risk of bias (ROB) assessment, a diverse range of bias risk across the evaluated studies was revealed. On average, nearly 44% of the studies were classified as having a low risk of bias. Approximately 28% of the studies fell into the category of “Some Concerns” risk of bias and 28% of the studies were identified as having a high risk of bias. A total of thirteen studies provided a comprehensive description of all domains as per the Cochrane Collaboration’s tool. Figure 2 and Figure S1 provide visual representations of ROB assessment of each study in every domain.

### Clinical outcomes following stem cell therapy

**Fig. 3:**
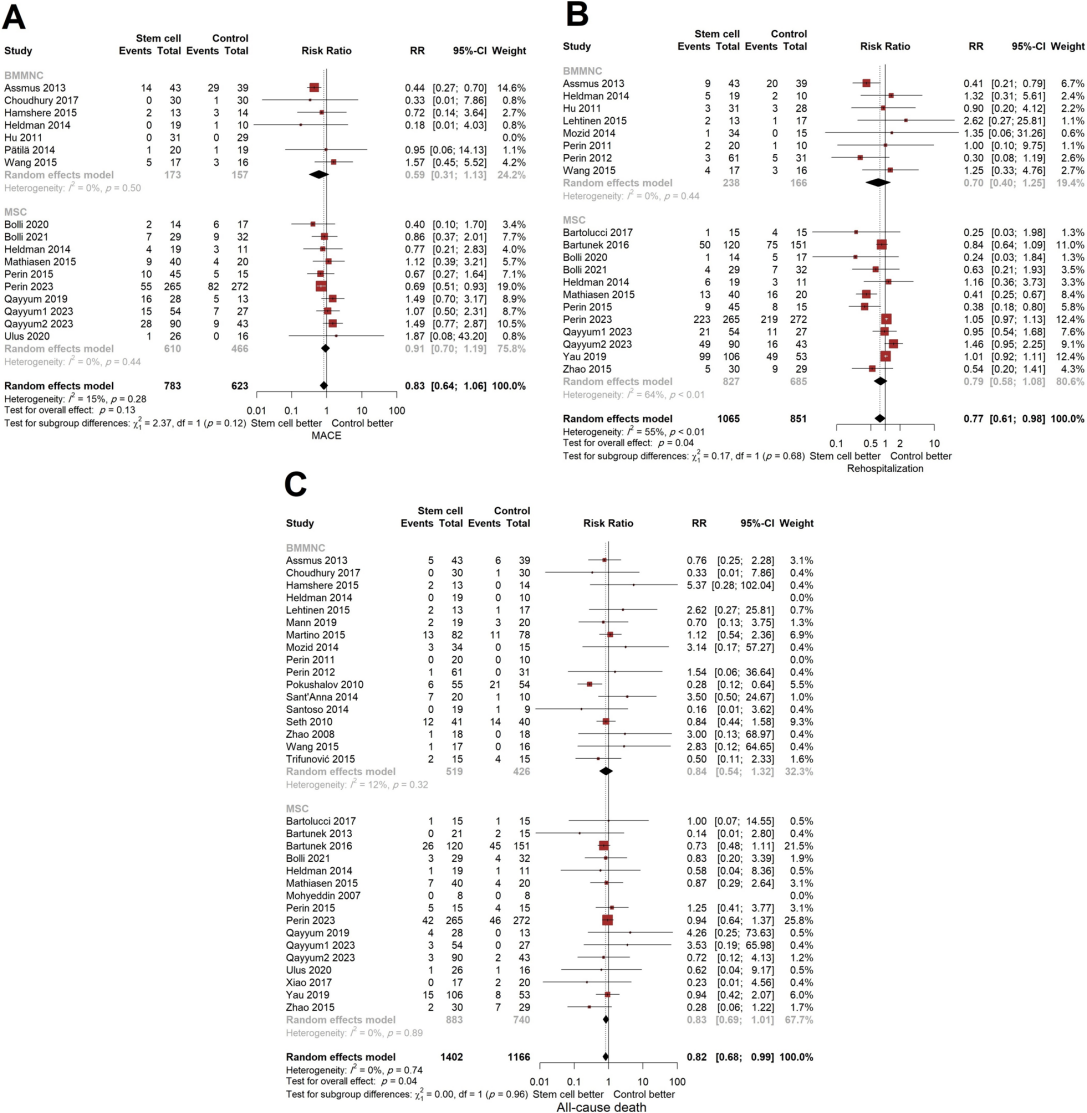
Forest plot demonstrating the comparison of stem cell transplantation compared with placebo stratified by the type of cell **A**: MACE, **B**: all-cause mortality, and C: rehospitalization for heart failure (MACE: major adverse cardiovascular events, RR: risk ratio, CI: confidence interval, BMMNC: bone-marrow mononuclear cell, MSC: mesenchymal stem cell)

A total of 33 studies (1402 patients in the stem cell group and 1166 patients in the control group) reported data regarding mortality. Pooled estimate showed a 18% decrease in the risk of all-cause mortality in the stem cell group compared with the controls (RR (95% CI) = 0.82 (0.68, 0.99), *p* = 0.04). Although the RR of mortality was marginally significant, BMMNC and MSC therapy could not decrease the risk of mortality in the longest follow-up period (BMMNC: RR (95% CI) = 0.84 (0.54, 1.32), MSC: RR (95% CI) = 0.83 (0.69, 1.01), between-subgroup *p* = 0.96). Stem cell therapy could not change the risk of long-term MACE compared to the controls (RR (95% CI) = 0.83 (0.64, 1.06), *p* = 0.13) and the subgroups were similar regarding risk reduction of MACE (BMMNC: RR (95% CI) = 0.59 (0.31, 1.13), MSC: RR (95% CI) = 0.91 (0.70, 1.19), between-subgroup *p* = 0.12). The transplantation of stem cells resulted in a marginally significant decrease in the risk of rehospitalization in long-term follow-up (RR (95% CI) = 0.77 (0.61, 0.98), *p* = 0.04) with no difference between subgroups (*p* = 0.68) (Fig. 3).

### Echocardiographic parameters

**Fig. 4:**
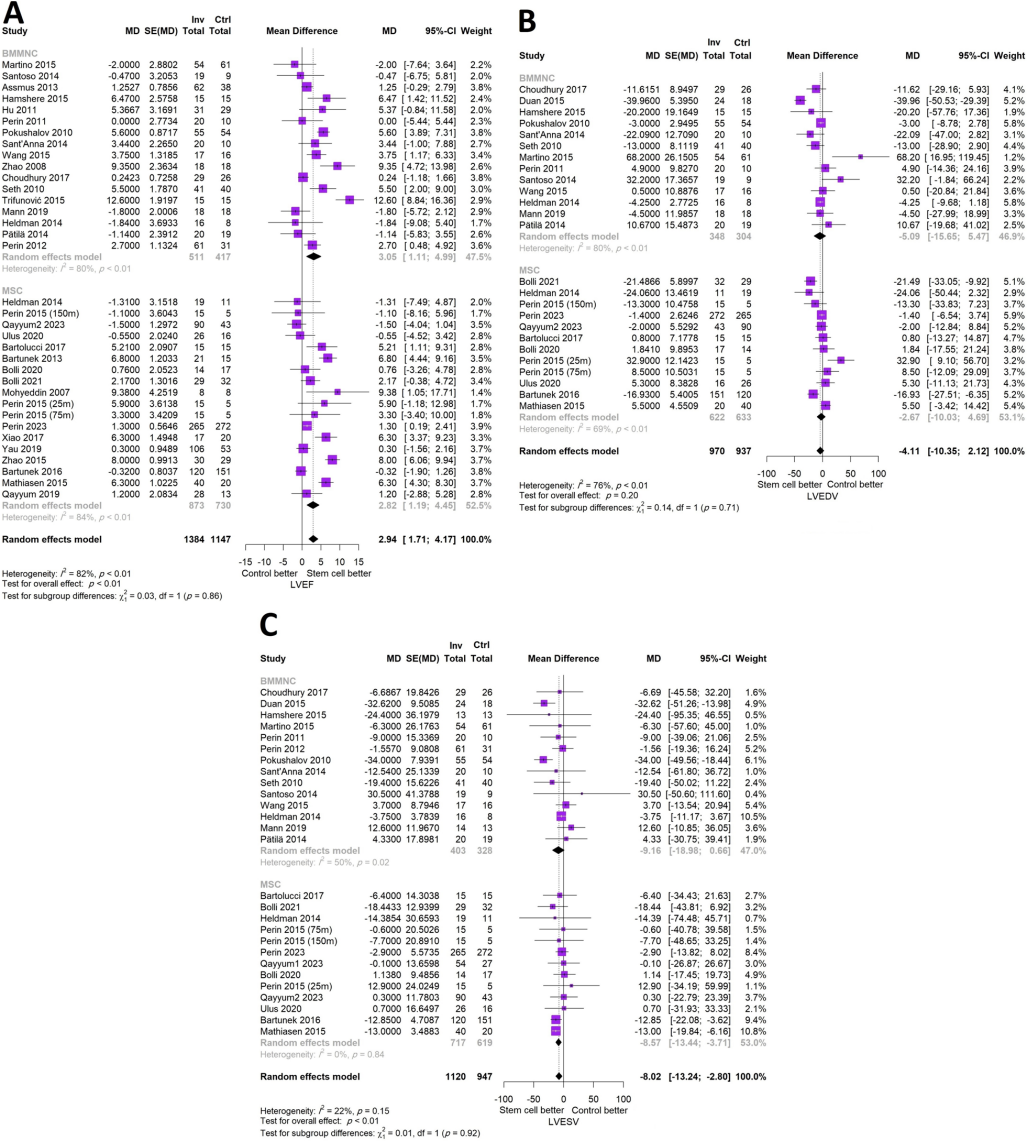
Forest plot demonstrating the comparison of stem cell transplantation compared with placebo stratified by the type of cell **A**: LVEF, **B**: LVEDV, and C: LVESV (LVEF: left ventricular ejection fraction, LVEDV: left ventricular end-diastolic volume, LVESV: left ventricular end-systolic volume, MD: mean difference, CI: confidence interval, BMMNC: bone-marrow mononuclear cell, MSC: mesenchymal stem cell)

After inclusion of 17 studies performing BMMNC therapy and 16 studies with MSC transplantation, stem cell transplantation resulted in a significant increase in LVEF compared with the control group (MD (95% CI) = 2.94% (1.71, 4.17), *p* < 0.001). Both subgroups of BMMNCs and MSCs were effective in increasing LVEF although there was not a statistically significant difference between the subgroups (BMMNC: MD (95% CI) = 3.05% (1.11, 4.99), MSC: MD (95% CI) = 2.82% (1.19, 4.45), *p* = 0.86). There was no statistically significant difference in LVEDV change following stem cell therapy compared with the control group (MD (95% CI) = -4.11 (-10.35, 2.12), *p* = 0.20) with no difference across subgroups (*p* = 0.71). Regarding the change in LVESV, transplantation of stem cells was concomitant with a statistically significant decrease in LVESV (MD (95% CI) = -8.02 (-13.24, -2.80), *p* < 0.001) mainly driven from MSC therapy rather than BMMNC transplantation (BMMNC: MD (95% CI) = -9.16 (-18.98, 0.66), MSC: MD (95% CI) = -8.57 (-13.44, -3.71), *p* = 0.92) (Fig. 4).

### 6-min walk test and brain natriuretic peptide

**Fig. 5:**
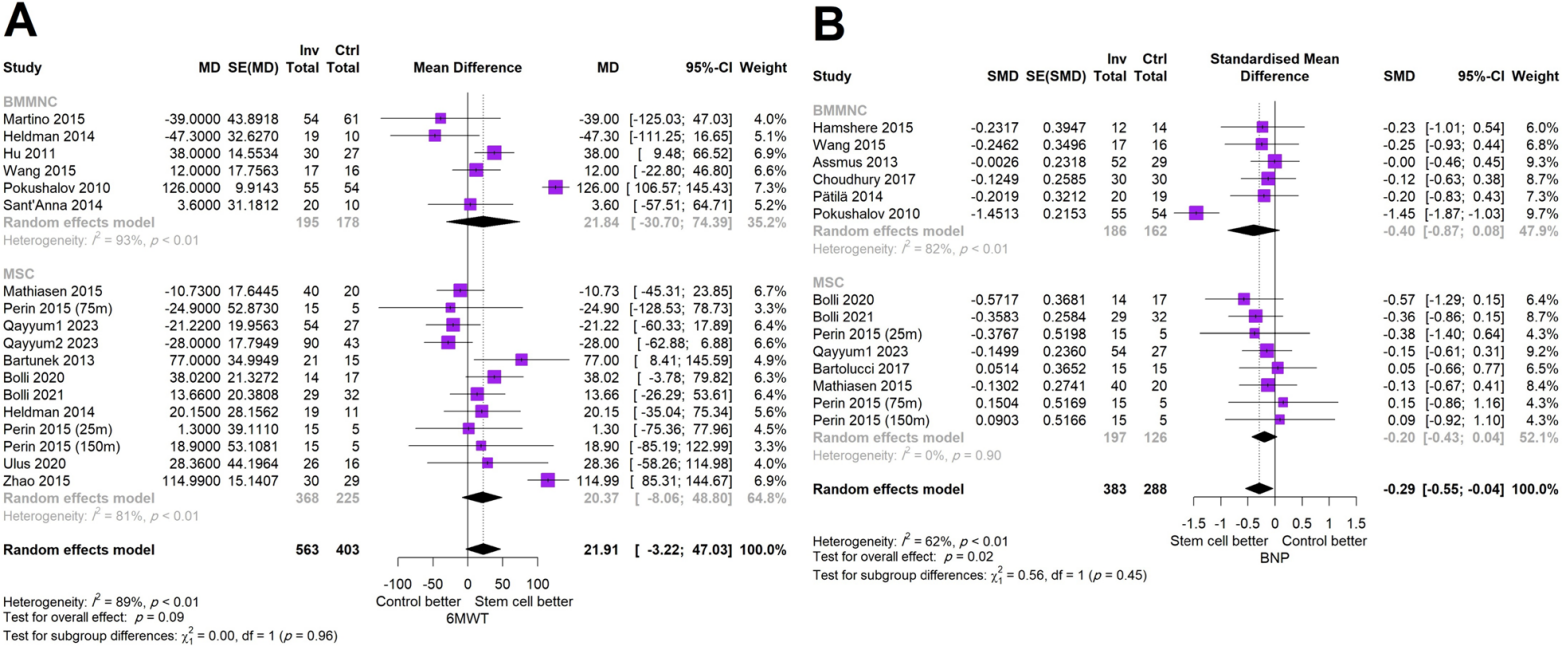
Forest plot demonstrating the comparison of stem cell transplantation compared with placebo stratified by the type of cell **A**: 6MWT and **B**: BNP (6MWT: 6-min walk test, BNP: B-type natriuretic peptide, SMD: standardized mean difference, MD: mean difference, CI: confidence interval, BMMNC: bone-marrow mononuclear cell, MSC: mesenchymal stem cell)

The results of the 6MWT from 18 studies were pooled and the estimate showed no statistically significant difference between stem cells and control group (MD (95% CI) = 21.91 (-3.22; 47.03), *p* = 0.09). No noticeable difference was also observed among the two types of stem cells (BMMNC: MD (95% CI) = 21.84 (-30.70, 74.39), MSC: MD (95% CI) = 20.37 (-8.06, 48.80), *p* = 0.96). After pooling the results of 14 studies reporting the changes in BNP, stem cell therapy led to a significant decrease in BNP levels compared with placebo (SMD (95% CI) = -0.29 (-0.55; -0.04), *p* = 0.02) although no difference was shown between BMMNCs (SMD (95% CI) = -0.40 (-0.87; 0.08)) and MSCs (SMD (95% CI) = -0.20 (-0.43; 0.04)) (Fig. 5).

### Route of stem cell delivery

The routes of injection included intracoronary, intramyocardial, through graft vessel during coronary artery bypass grafting (CABG), transendocardial, and intravenous. Regarding LVEF, all of the injection routes including intracoronary, intramyocardial, and transendocardial could improve the ventricular function significantly. In terms of MACE, the transendocardial injection of stem cells was superior compared with other cell types. For other outcomes, there was no considerable difference among subgroups (Figure S2-S9).

### ICEMAN credibility assessment

According to Table 2, all the included variables were assessed by the ICEMAN instrument. Except for one of the studied parameters (LVESV), the endpoints of interest were unlikely to have different magnitude of effect across the two subgroups (BMMNC and MSC) and therefore, they were rated as “likely no effect modification”. For LVESV, the MSC subgroup showed superior results compared with BMMNC as BMMNCs could not demonstrated a statistically significant change in LVESV compared with their controls.

**Table 2:** ICEMAN criteria for meta-analysis of randomized controlled trials

Variable	Q1	Q2	Q3	Q4	Q5	Q6	Q7	Q8	Overall rating	Interpretation
MACE	Completely between	NA	Rather large	Unclear	Chance a very likely explanation (0.12)	Definitely yes	Definitely yes	NA	Maximum usually low	Likely no effect modification. Use overall effect for each subgroup but note remaining uncertainty
All-cause mortality	Completely between	NA	Large	Unclear	Chance a very likely explanation (0.96)	Definitely yes	Definitely yes	NA	Maximum usually low	Likely no effect modification. Use overall effect for each subgroup but note remaining uncertainty
Rehospitalization	Completely between	NA	Rather large	Unclear	Chance a very likely explanation (0.68)	Definitely yes	Definitely yes	NA	Maximum usually low	Likely no effect modification. Use overall effect for each subgroup but note remaining uncertainty
LVEF	Completely between	NA	Large	Unclear	Chance a very likely explanation (0.86)	Definitely yes	Definitely yes	NA	Maximum usually low	Likely no effect modification. Use overall effect for each subgroup but note remaining uncertainty
LVEDV	Completely between	NA	Large	Unclear	Chance a very likely explanation (0.71)	Definitely yes	Definitely yes	NA	Maximum usually low	Likely no effect modification. Use overall effect for each subgroup but note remaining uncertainty
LVESV	Completely between	NA	Large	Definitely yes	Chance a very likely explanation (0.92)	Definitely yes	Definitely yes	NA	Maximum usually moderate	Likely effect modification. Use separate effects for each subgroup but note remaining uncertainty
6MWT	Completely between	NA	Rather large	Probably yes	Chance a very likely explanation (0.96)	Definitely yes	Definitely yes	NA	Maximum usually low	Likely no effect modification. Use overall effect for each subgroup but note remaining uncertainty
BNP	Completely between	NA	Rather large	Probably yes	Chance a very likely explanation (0.45)	Definitely yes	Definitely yes	NA	Maximum usually low	Likely no effect modification. Use overall effect for each subgroup but note remaining uncertainty

Q1, Is the analysis of effect modification based on comparison within rather than between trials? Q2, for within-trial comparisons, is the effect modification similar from trial to trial? Q3, for between-trial comparisons, is the number of trials large? Q4, Was the direction of the effect modification correctly hypothesized priori? Q5, does a test for interaction suggest that chance is an unlikely explanation of the apparent effect modification? Q6, Did the authors test only a small number of effect modifiers? Q7, Did the authors use a random effects model? Q8, If the effect modifier is a continuous variable, were arbitrary cut points avoided?

## Discussion

In this systematic review and meta-analysis, which included 35 clinical trials, effectiveness of stem cell transplantation therapy using mesenchymal stem cells (MSCs) or bone marrow-derived mononuclear cells (BMMNCs) in heart failure patients was assessed and compared using different clinical outcomes and echocardiographic indices. The primary findings of this meta-analysis are as follows: (1) The combined effect from these clinical trials supports the hypothesis that both MSC and BMMNC therapy are effective in increasing LVEF, with estimates slightly favoring BMMNCs (MD of 3.05% vs 2.82%), however there is no statistically significant difference between the effects of the interventions (p = 0.86). (2) The improvement in LVEF was not translated to superior results of stem cells compared with placebo regarding MACE although a marginally significant decrease in the risk of all-cause mortality and rehospitalization was noted compared with placebo. (3) Other secondary outcomes and indices extracted from the studies were also pooled to make a better comparison between the efficacy of MSCs and BMMNCs, however no statistical difference was observed between MSCs and BMMNCs groups in any of the indices (p-value of interaction ranging from 0.12 to 0.96). Based on our current understanding, although there have been meta-analyses which compared these cell types as a part of their subgroup analysis [56], analyses performed may have been underpowered, with few studies in each group. Subsequently, this meta-analysis represents a more inclusive investigation comparing the impact of these two distinct cell therapies in patients suffering from heart failure. Our results demonstrated that although the stem cells may result in a significant increase in LVEF, it may not cause a decrease in the rates of long-term MACE and future randomized trials powered to assess clinical outcomes are needed to evaluate the efficacy of stem cells compared with placebo in HF patients.

Prior studies have shown that MSC treatment exhibits remarkable efficacy in enhancing echocardiographic parameters among patients with acute myocardial infarction [57]. Similarly, BMMNC treatment has demonstrated comparable effects, showing promise as a viable therapeutic approach for patients with heart failure [58]. A meta-analysis done by Kalou et al. [59], was conducted to determine the efficacy of MSCs for heart failure treatment. Results from that study showed that treatment with MSCs resulted in a significant improvement in LVEF, with an increase of 4.43% when compared to the control groups [59]. Another systematic review and meta-analysis done by Fan et al. [60], made a pooled analysis of several indices to investigate efficacy of MSCs therapy in systolic heart failure. According to their study, MSCs therapy increased LVEF by 5.25% compared to the placebo group. The TAC-HFT trial was a randomized study including a direct comparison of MSCs with BMMNCs showing better function of MSCs in terms of reducing the infarct size and improving regional myocardial infarction compared with BMMNCs and placebo but no difference regarding LVEF change[24]. Regarding the subject of LVEF, the findings of these studies align with the results of our meta-analysis. An interesting finding in our analysis was that although both cells were proved to increase LVEF, there was no superior type of cell and this was further confirmed with assessing the credibility of the results using ICEMAN tool. It should be mentioned that a meta-analysis comparing MSCs and BMMNCs showed that MSCs may have better efficacy regarding LVEF in the setting of acute myocardial infarction [61] but according to this study, this was not the case for patients with chronic heart failure.

The findings from our meta-analysis suggest that treatment with MSCs and BM-MNCs is linked to a notable enhancement in LVEF and LVESV, as pooled mean differences of LVEF and LVESV both excluded the null zone and were statistically significant regardless of the type of cell therapy. However, overall mean difference of LVEDV was not statistically significant in any of the subgroups. A systematic review and meta-analysis carried out by Wang et al. [62], also found similar outcomes for these three echocardiographic parameters following stem cell transplantation. This consistency of results further substantiates the notion that, for reasons yet to be fully understood and further investigated, the transplantation of MSCs or BM-MNCs appears to improve ventricular function by increasing LVEF and reducing LVESV without having any considerable effect on LVEDV.

An earlier meta-analysis conducted by Fisher et al. concluded that cell therapy could decrease the risk of all-cause mortality with a medium grade efficacy [56]. However, our analysis does not support the previous finding. The combined risk ratio of all cell therapies (RR 0.82, 95% CI 0.68 to 0.99) for all-cause mortality was statistically significant and excluded the null zone but indicated a small grade efficacy that is borderline trivial. On the other hand, the combined effect size of BMMNCs (RR 0.84, 95% CI 0.54 to 1.32) and MSCs therapy (RR 0.83, 95% CI 0.69 to 1.01) suggests that the impact of this intervention on all-cause mortality is not even statistically significant. This rather considerable discrepancy between our study and Fisher et al., might be due to the larger number of participants in our study.

The pooled analysis on major adverse cardiac events (MACE) rates yielded no statistically significant difference between any of the cell therapy groups and the placebo, nor was there a significant difference among the cell therapy groups themselves. However, looking at the combined effect size from all studies, stem cell therapy can have a marginal effect in reducing the rate of rehospitalization (RR 0.77, 95% CI 0.61 to 0.98) in patients receiving it compared to placebo. Also, the pooled estimate showed a marginally significant risk reduction of all-cause death by 18% compared with placebo. This is the first meta-analysis comparing the clinical outcomes between MSCs and BMMNCs using randomized trials. Our results are consistent with a previous meta-analysis that showed MSCs could not reduce the risk of adverse clinical events[60]. The mentioned study showed better outcomes regarding readmission in comparison to the control group after inclusion of five trials. It is noteworthy that our analysis included 12 studies in the MSC subgroup which may have yielded more reliable results compared to previous ones and showed no difference efficacy compared with placebo. Furthermore, although the contemporary evidence has shown no superior efficacy of stem cells compared with placebo in HF patients, the majority of the trials were not powered to assess the clinical outcomes and this may have led to the non-significant results. Also, a previous meta-analysis has shown the better cardiovascular outcomes after BMMNC therapy in the setting of acute myocardial infarction[63]. Future well-designed and large-scale trials should primarily focus on clinical outcomes to assess if improvement in echocardiographic indices can be translated into favorable clinical outcomes or not.

Previous studies have presented 6MWT and BNP as potential prognostic factors in patients with heart failure. It has been demonstrated that each 100 pg/mL increase in BNP levels can increase the hazard of adverse outcomes by 14% [64, 65]. Our results showed that stem cell therapy can cause a more considerable decrease in serum BNP compared with no stem cell transplantation although no apparent difference was noted between BMMNCs and MSCs. Regarding the distance walked during 6MWT, stem cell group could increase the distance of 6MWT by 21.9 m but this difference was marginally non-significant. Also, there was no difference among the stem cell groups. It can be assumed that although stem cell transplantation may result in improvements in prognostic factors (as demonstrated by reduction in BNP levels and marginally non-significant improvement in 6MWT), there may be no superior type of stem cell in this regard and these results present opportunities for future investigations in randomized trials.

The primary limitation of this meta-analysis, similar to any review, lies in the fact that there is a lack of uniformity across studies in terms of the patient population, the implementation of the intervention, and the definitions of outcomes. Another limitation of our study was the fact that the quality of the studies varied. In the majority of the trials, the process of randomization was executed appropriately. However, there were three studies that did not clearly indicate that their data analysis was conducted in accordance with the intention-to-treat principle. This omission could potentially result in an overestimation of the treatment effect in these particular trials. Moreover, head-to-head comparison of cell types was not performed in any of the studies except one, hence, the only way to compare MSCs vs. BMMNCs was through subgroup analysis and the test to spot any difference between subgroups. Also, many of the included trials were focused on echocardiographic indices rather than long-term clinical outcomes and hence, they were not powered to compare clinical outcomes.

## Conclusion

In conclusion, we showed that both MSCs and BMMNCs were effective in terms of improving LVEF and decreasing BNP compared with placebo in patients with chronic heart failure and no apparent difference was observed between the two types of cells. Transplantation of MSCs and BMMNCs was not translated into better MACE but a marginally significant improvement in rehospitalization and all-cause mortality at long-term. Future well-designed randomized trials are warranted to further investigate the possible benefits of stem cell therapy in patients with heart failure.

### Supplementary Information

Additional file 1.

## Data Availability

The data underlying this article will be shared on reasonable request from the corresponding author.

## Abbreviations

IL-1β: Interleukin-1β
TNF-α: Tissue necrosis factor-α
VEGF: Vascular endothelial growth factor
HF: Heart failure
MSC: Mesenchymal stem cell
BMMNC: Bone-marrow mononuclear cell
PRISMA: Preferred reporting items for systematic reviews and meta-analyses
RCT: Randomized controlled trial
MACE: Major adverse cardiovascular events
LVEF: Left ventricular ejection fraction
LVEDV: Left ventricular end-diastolic volume
LVESV: Left ventricular end-systolic volume
RR: Risk ratio
CI: Confidence interval
MD: Mean difference
ICEMAN: Instrument to assess the credibility of effect modification analyses
CABG: Coronary artery bypass grafting
