# Supplementary material for: Comparative effectiveness of mesenchymal stem cell versus bone-marrow mononuclear cell transplantation in heart failure: a meta-analysis of randomized controlled trials
Source: Stem Cell Res Ther. 2024 Jul 6;15:202. doi: 10.1186/s13287-024-03829-7 (PMC11227704; doi:10.1186/s13287-024-03829-7)
Supplement: Supplementary file 1 — Additional file 1. [file 13287_2024_3829_MOESM1_ESM.docx]

**Table S1: Search strategy**

| **PUBMED** |
| --- |
| ((bone marrow cell) OR (mononuclear cell) OR (mesenchymal stem cell)) AND ((heart failure) OR (cardiomyopathy)) NOT (review):  (("mesenchymal stem cells"[MeSH Terms] OR "MSCs"[All Fields] OR ("bmsc s"[All Fields] OR "bmscs"[All Fields]) OR (("umbilical cord"[MeSH Terms] OR ("umbilical"[All Fields] AND "cord"[All Fields]) OR "umbilical cord"[All Fields]) AND ("mesenchymal stem cells"[MeSH Terms] OR ("mesenchymal"[All Fields] AND "stem"[All Fields] AND "cells"[All Fields]) OR "mesenchymal stem cells"[All Fields])) OR ("Adipose-derived"[All Fields] AND ("stem cells"[MeSH Terms] OR ("stem"[All Fields] AND "cells"[All Fields]) OR "stem cells"[All Fields])) OR (("umbilical cord"[MeSH Terms] OR ("umbilical"[All Fields] AND "cord"[All Fields]) OR "umbilical cord"[All Fields]) AND ("stem cells"[MeSH Terms] OR ("stem"[All Fields] AND "cells"[All Fields]) OR "stem cells"[All Fields])) OR ("ucmsc"[All Fields] OR "ucmscs"[All Fields]) OR "ADSCs"[All Fields] OR ("bone marrow cells"[MeSH Terms] OR ("bone"[All Fields] AND "marrow"[All Fields] AND "cells"[All Fields]) OR "bone marrow cells"[All Fields] OR ("bone"[All Fields] AND "marrow"[All Fields] AND "cell"[All Fields]) OR "bone marrow cell"[All Fields] OR (("mononuclear"[All Fields] OR "mononuclears"[All Fields]) AND ("cells"[MeSH Terms] OR "cells"[All Fields] OR "cell"[All Fields])))AND ("heart failure"[MeSH Terms] OR ("heart"[All Fields] AND "failure"[All Fields]) OR "heart failure"[All Fields] OR ("cardiomyopathie"[All Fields] OR "cardiomyopathies"[MeSH Terms] OR "cardiomyopathies"[All Fields] OR "cardiomyopathy"[All Fields]))) NOT ("review"[Publication Type] OR "review literature as topic"[MeSH Terms] OR "review"[All Fields]) |
| **EMBASE** |
| ('bone marrow mononuclear cell'/exp OR 'bone marrow mononuclear cell' OR (('bone' OR 'bone'/exp OR bone) AND ('marrow' OR 'marrow'/exp OR marrow OR 'mesenchymal stem cells'/exp OR 'mesenchymal stem cells') AND mononuclear AND ('cell' OR 'cell'/exp OR cell))) AND ('heart failure':ti,ab,kw OR cardiomyopathy:ti,ab,kw) |
| **SCOPUS** |
| ( ( TITLE-ABS-KEY ( heart AND failure ) OR TITLE-ABS-KEY ( cardiomyopathy ) ) ) AND ( ( TITLE-ABS-KEY ( bone AND marrow AND mononuclear AND cell ) OR TITLE-ABS-KEY ( mesenchymal AND stem AND cells ) OR TITLE-ABS-KEY ( mscs ) OR TITLE-ABS-KEY ( adipose-derived AND stem AND cells ) OR TITLE-ABS-KEY ( umbilical AND cord AND mesenchymal AND stem AND cells ) OR TITLE-ABS-KEY ( ucmscs ) OR TITLE-ABS-KEY ( adscs ) ) ) |

**Figure S1:** Traffic-light plot of the included trials


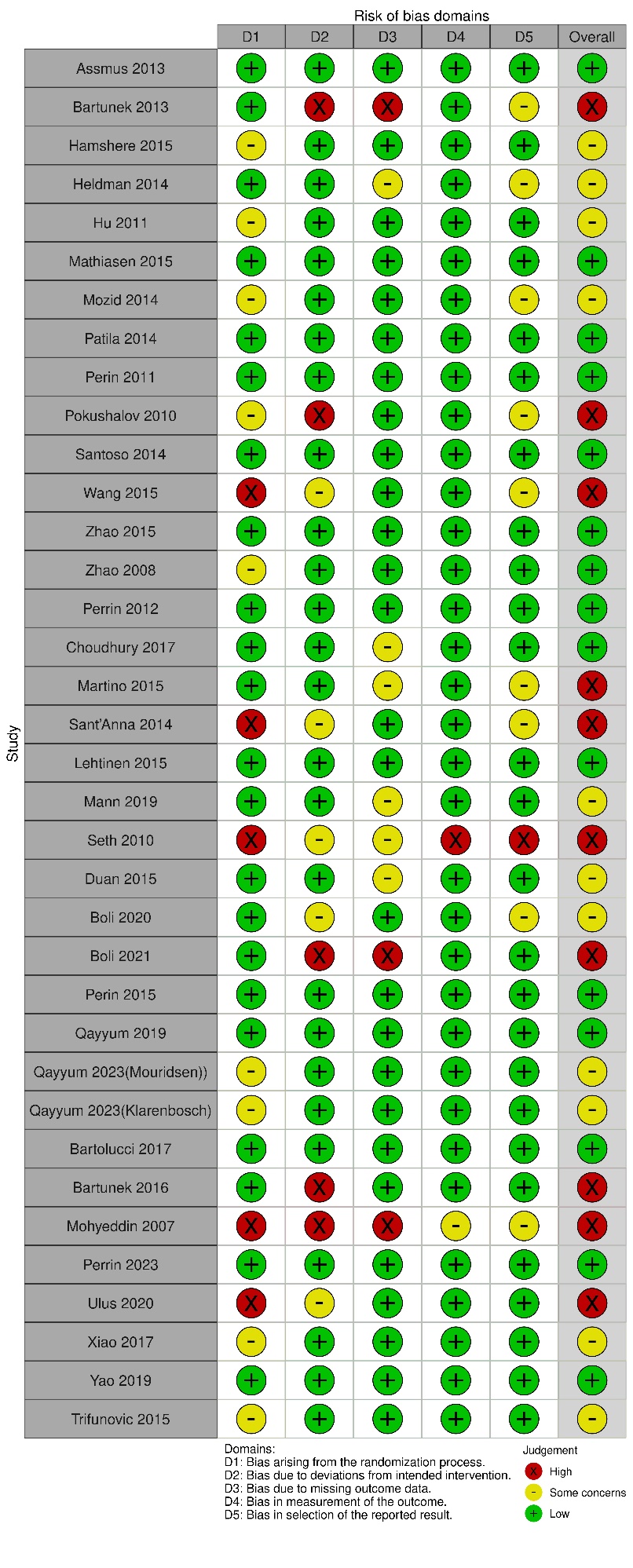


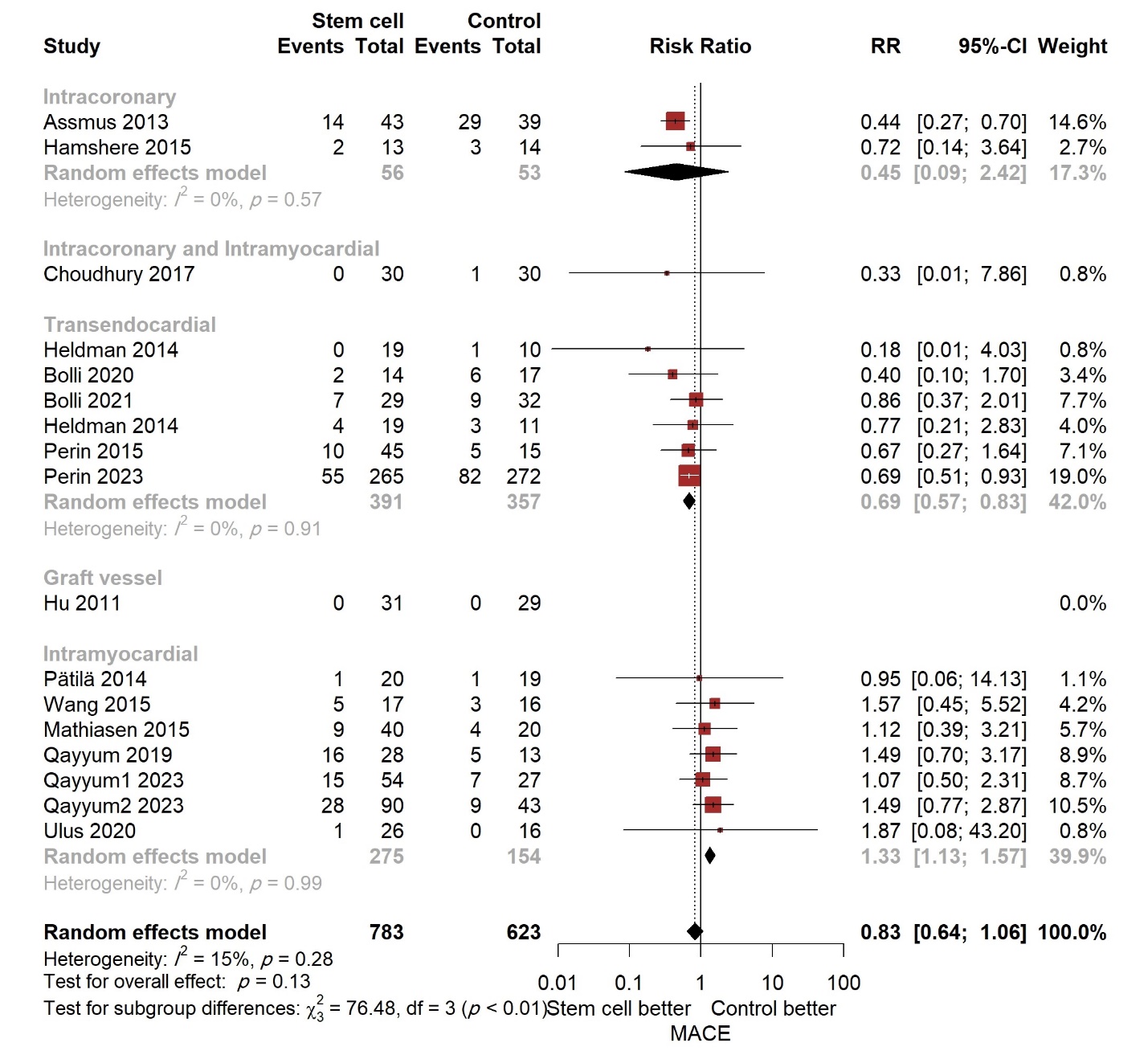


**Figure S2:** Forest plot comparing MACE based on route of injection


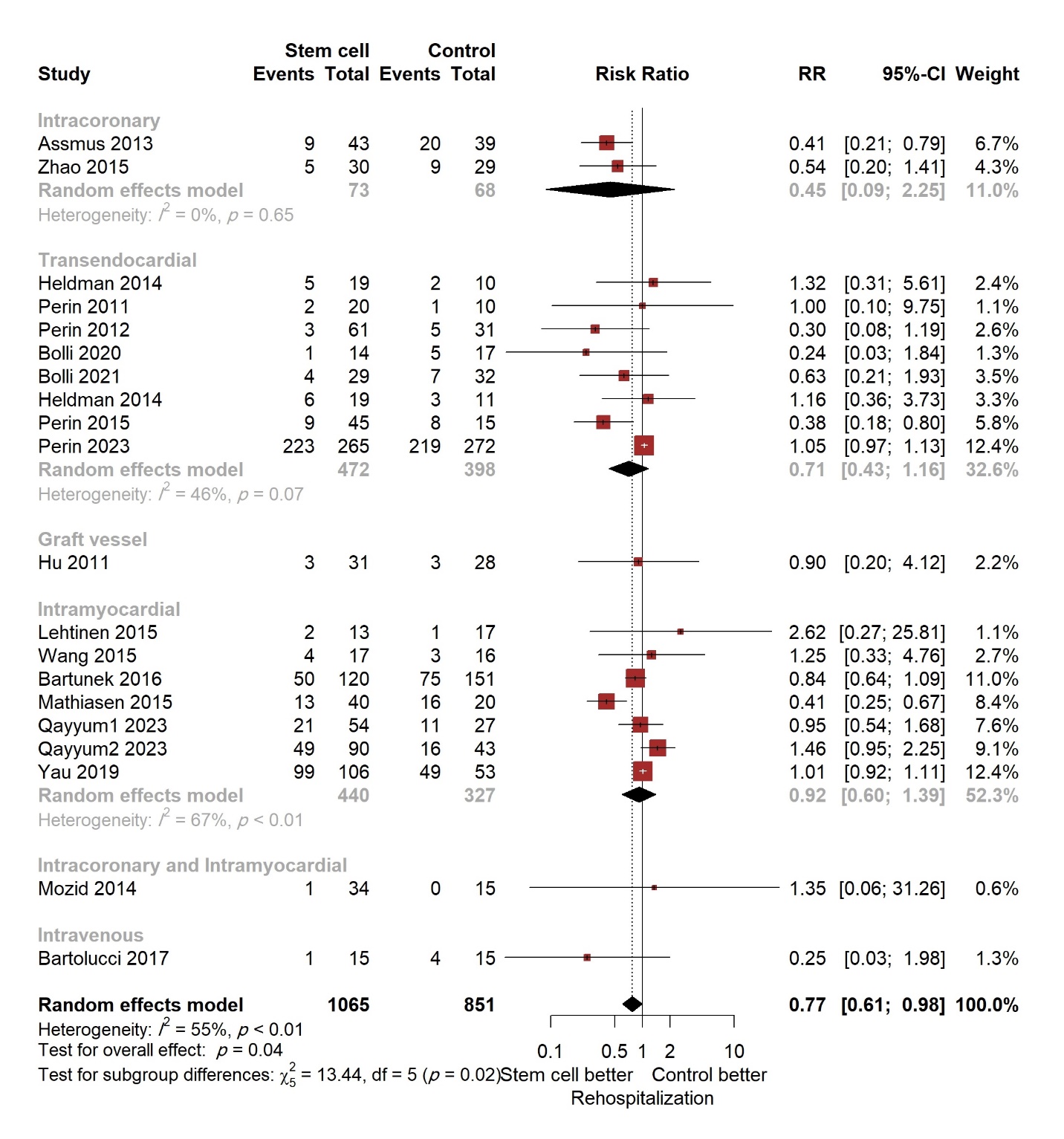


**Figure S3:** Forest plot comparing rehospitalization based on route of injection


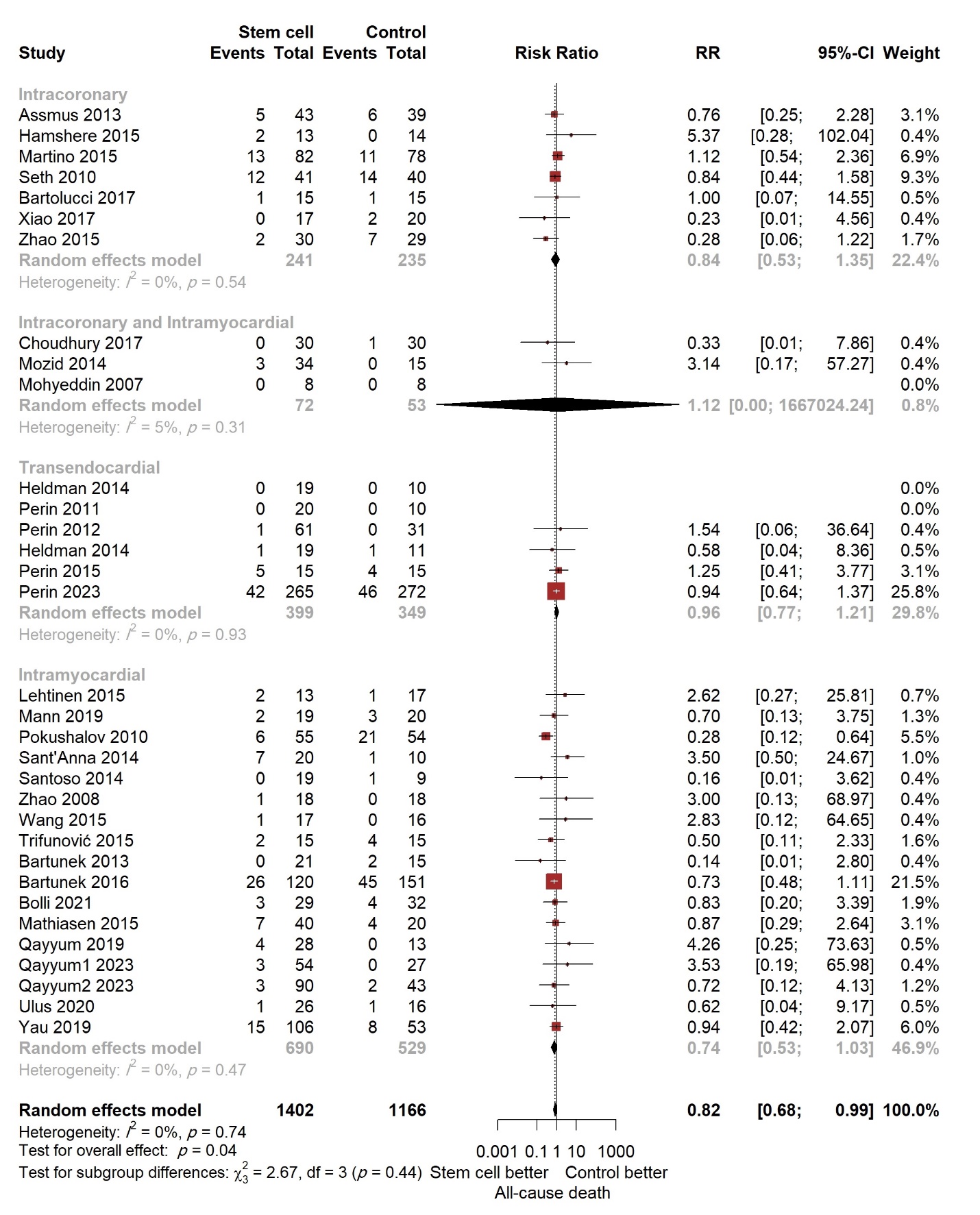


**Figure S4:** Forest plot comparing all-cause death based on route of injection


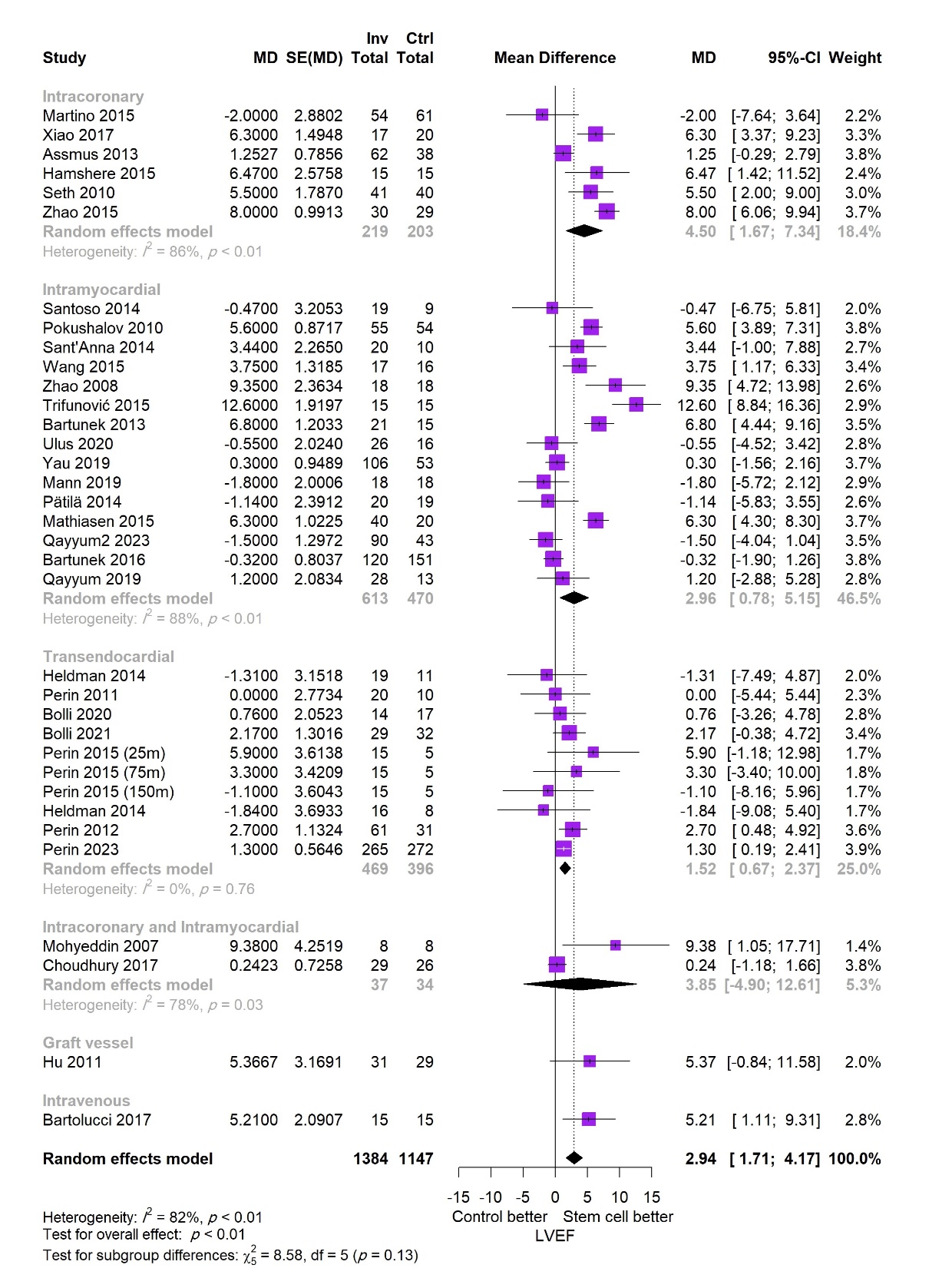


**Figure S5:** Forest plot comparing LVEF based on route of injection


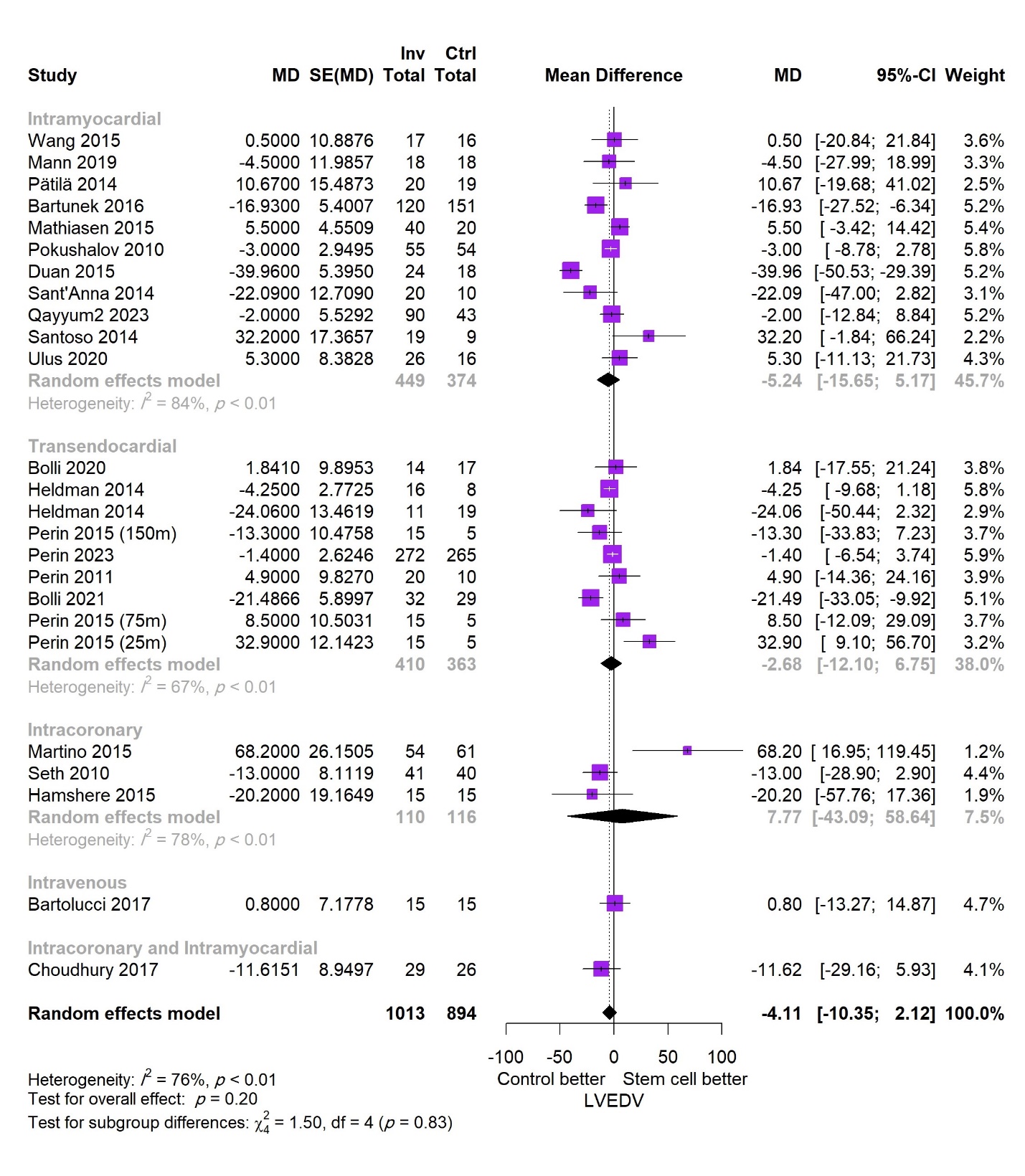


**Figure S6:** Forest plot comparing LVEDV based on route of injection


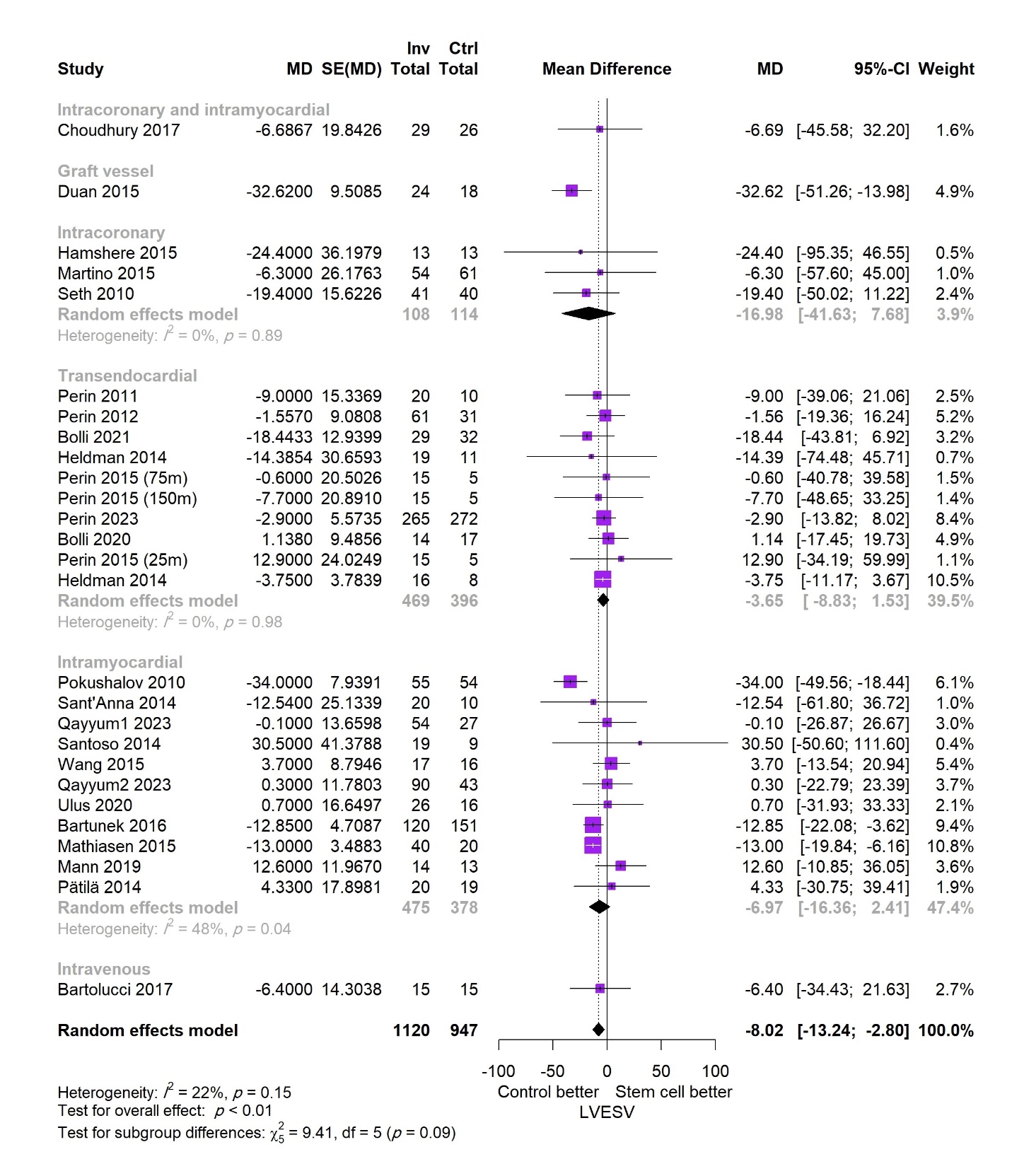


**Figure S7:** Forest plot comparing LVESV based on route of injection


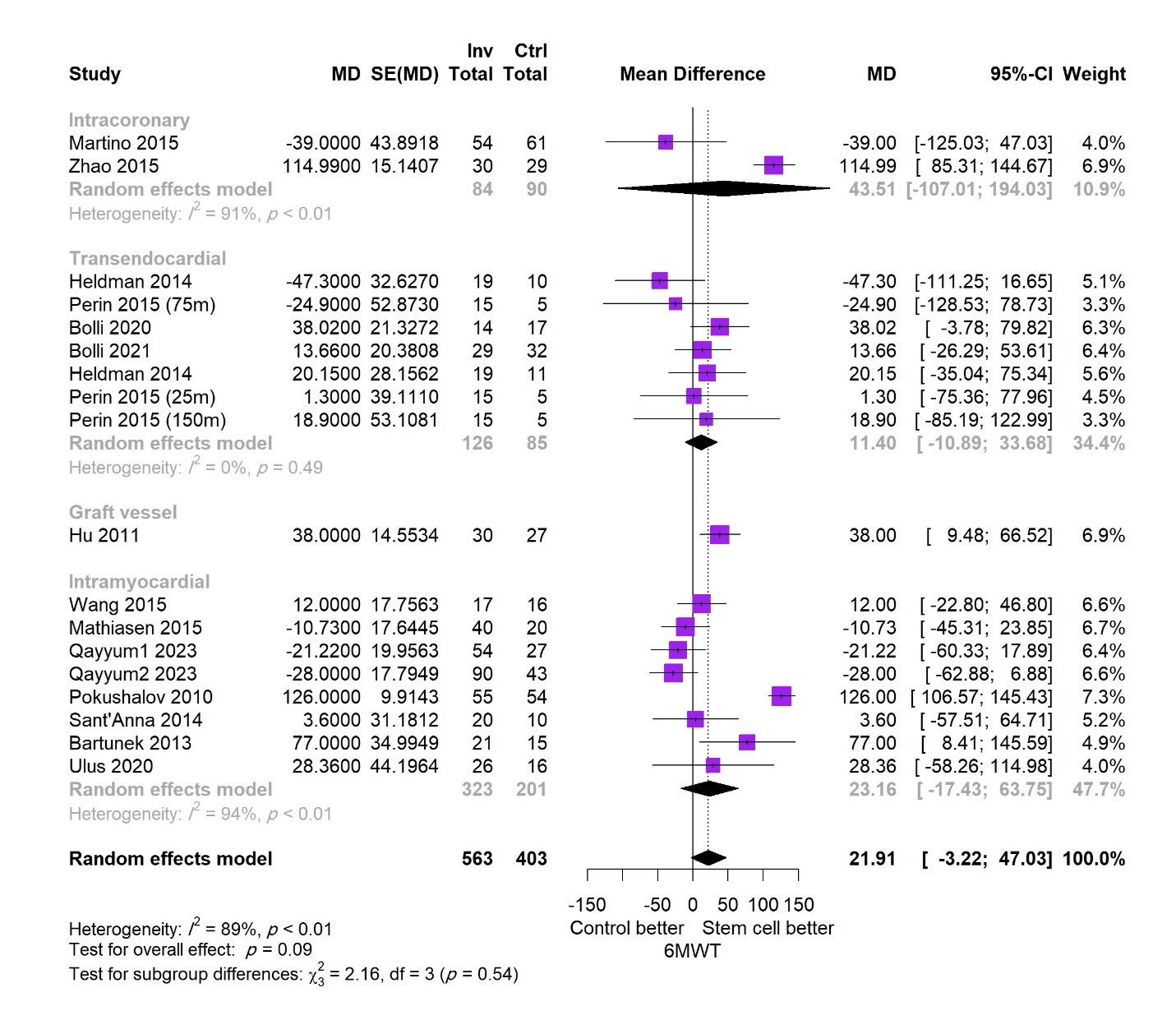


**Figure S8:** Forest plot comparing 6MWT based on route of injection


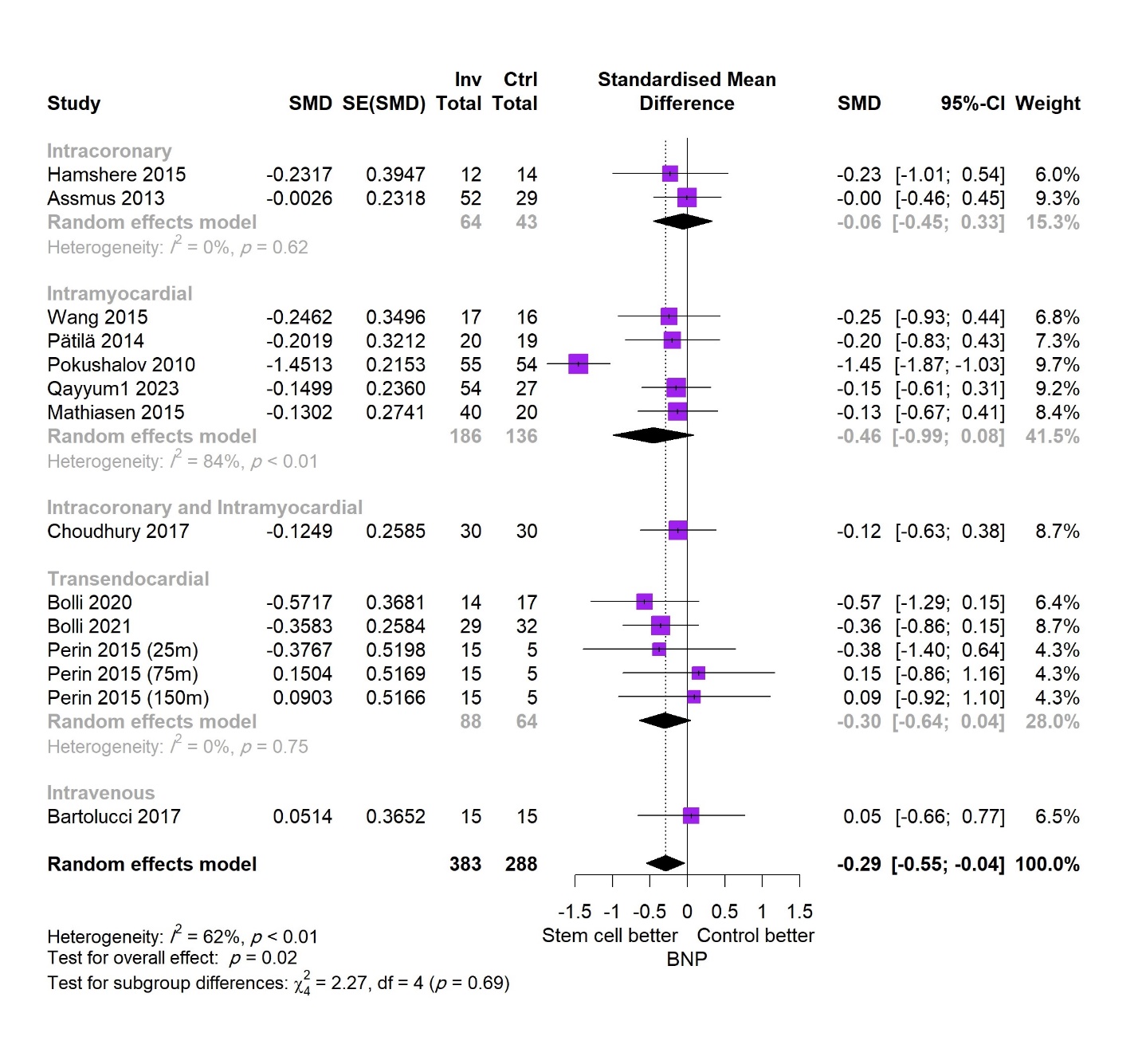


**Figure S9:** Forest plot comparing BNP based on route of injection
